# Barriers to adequate nutrition care for child malnutrition in a low-resource setting: Perspectives of health care providers

**DOI:** 10.3389/fpubh.2023.1064837

**Published:** 2023-03-09

**Authors:** Ghada Wahby Elhady, Sally kamal Ibrahim, Enas S. Abbas, Ayat Mahmoud Tawfik, Shereen Esmat Hussein, Marwa Rashad Salem

**Affiliations:** ^1^Public Health and Community Medicine Department, Faculty of Medicine, Cairo University, Manial, Cairo, Egypt; ^2^Pediatric Department, Faculty of Medicine, Cairo University, Manial, Cairo, Egypt; ^3^Pediatric Clinical Nutrition Department, National Nutrition Institute, Cairo, Egypt; ^4^Public Health and Community Medicine Department, Faculty of Medicine, Port Said University, Port Said, Egypt

**Keywords:** malnutrition, low-resource healthcare settings, skill-based training, updated standard of practice, guidelines, nutritional supplements, patient education materials

## Abstract

**Introduction:**

Several studies in developing countries found that more need-based training is required for health care providers (HCPs) in child malnutrition management.

**Methods:**

An exploratory cross-sectional study was conducted to explore barriers to providing adequate nutrition care as perceived by the healthcare providers (HCPs) in the child malnutrition clinic at a Children's University Hospital in Egypt. Participants were selected using the purposive sampling technique. Five out of seven HCPs in the clinic were included (two male physicians, one female physician, and two female nurses). Qualitative data were collected through in-depth interviews. The interview guide consisted of semi-structured open-ended questions. Quantitative data were the resulting scores from the scoring system used to assess the understandability and actionability of the patient education materials (PEMs) that are available in the clinic. The Patient Education Materials Assessment Tool for Printable Materials (PEMAT-P) for the scoring. Statistical analysis: The thematic content analysis technique was employed for qualitative data. The percent score was generated for the PEM actionability and understandability for quantitative data.

**Results:**

Most common child malnutrition conditions encountered by HCPs were nutritional deficiencies. Barriers to the delivery of adequate nutrition care to children were physician-centered: limited nutrition education in the medical school, health system-centered: an insufficient number of HCPs, lack of nutritional supplements, lack of patient education materials (PEMs) that suit the characteristics of the served community, lack of updated standard of practice (SOP) and guidelines, inadequate nutrition training of HCPs, and insufficient time for each patient, and caregivers-centered: the low socioeconomic status and false cultural, nutritional beliefs.

**Conclusion:**

There are different barriers to adequate nutrition care for child malnutrition in low-resource healthcare settings. Mainly nutritional deficiencies. Most of the barriers were health system-related in the form of insufficient resources (shortage of workforce; concerning the high caseload, nutritional supplements, and PEMs) and inadequate management of resources (lack of skill-based training, lack of updated SOP and guidelines, and lack of properly designed PEMs that facilitate communication with the target caregivers).

## Introduction

Malnutrition during infancy and childhood may lead to impaired growth, delayed and improper social and cognitive development, low academic achievement, and later in life, reduced productivity (1).

Globally, malnutrition is still a significant cause of death and disease among children, especially those under 5 years (U5Y) of age. Undernutrition type of malnutrition is associated with 45% of child deaths.^1^ Most of those deaths take place in low- and middle-income countries that also have rising rates of childhood obesity (see text footnote 1).^2^ In 2020, 38.9 million children U5Y of age were overweight worldwide, 45.4 million were wasted, and 149.2 million were stunted (see text footnote 2). Stunting, which is defined by the World Health Organization (WHO) as a height that is more than two standard deviations below the median child's growth standards, is largely irreversible^3^ and associated with extremely high health and economic costs^4^ (2). Stunting is the impact of chronic malnutrition afflicting the child during the first 1,000 days of life. The number of stunted children is declining in all world regions except Africa (see text footnote 4).

Children's nutritional status is a powerful and sensitive indicator for assessing child health, food security, and the need to improve economic, environmental, and health policies (see text footnote 1). Although Egypt has achieved remarkable progress in child health in the past two decades, the country's U5Y child mortality rate in 2013 was below the Millennium Development Goal (MDG) 44 (see text footnote 4), (2) and far below Sustainable Development Goal (SDG) 2 (3)^5^. The prevalence of child malnutrition in Egypt is predominantly high, with 11% of infants born with a low birth weightweight (see text footnote 2), and 9.5% of children U5Y of age are underweight, which is higher than the average for Africa (6.0%), and 22% are stunted, which is the largest prevalence of stunting in the Middle East (see text footnote 4), (2, 3). Furthermore, stunted growth is a public health problem that has persisted in Egypt for a long time, as reported in EDHS 2014 (percent of stunted U5Y children was reported as 23, 23, 29, and 22% in the years 2000, 2005, 2008, and 2014, respectively) (3).

Educating the caregivers of children has been proven in many studies to not only increase their knowledge but also improve the health outcomes of these children (4–6). A systematic review to explore factors associated with successful nutrition education interventions for children showed that engaging parents through face-to-face education and identification of specific child's or parent's behaviors that needed to be modified were fundamental factors (7). Thus, the role of health care providers (HCPs) in educating caregivers is crucial to overcoming child malnutrition (8). Several studies in developing countries found that there is a need for more in-service, need-based, and skill-based training of HCPs involved in child malnutrition management.

The main objective of the present study was to explore barriers to providing adequate nutrition care services in the child malnutrition clinic to inform future service delivery strategies for managing child malnutrition, particularly in low-resource healthcare settings.

## Materials and methods

### Design and context of the study

The current study is a clinic-based exploratory cross-sectional study that used a qualitative approach. The study was conducted in the Center of Social and Preventive Medicine (CSPM) Malnutrition Clinic, Faculty of Medicine, Cairo University, Egypt, among HCPs (physicians and nurses) of the clinic.

The study was performed in accordance with the Consolidated Criteria for Reporting Qualitative Research CORE-Q (9).

### Sampling technique and sample size

Participants were selected *via* a purposive sampling technique (10). The interviews were continued until they reached data saturation, where no new themes, subthemes, or explanations emerged from the interviews (11). ***Eligibility criteria*** were any HCPs (physicians and nurses) working in the CSPM malnutrition clinic during the study duration who were willing to participate. Out of seven HCPs, participants were five HCPs (two male physicians, one female physician, and two female nurses) because no new data were added starting from the third interview and saturation was achieved at the fifth interview. The interviewed physicians graduated between 1995 and 2001, and the interviewed nurses graduated in 1985 and 1990. One physician was a general practitioner; the other two were continuing their postgraduate studies in family medicine and pediatrics, respectively. The median number of working years mentioned by the HCPs was 6 years, ranging from 8 to 20 years.

#### Data types and collection tools

***Qualitative data*** were collected through in-depth, audiotaped face-to-face interviews (each lasting up to 45 min) conducted by one of the researchers, who has experience conducting interviews and has worked on many qualitative studies. The interview guide (Appendix 1), which was pilot-tested beforehand, consisted of semi-structured, open-ended questions. The interview guide was developed following systematic literature reviews (3, 4, 6, 8, 12). A trained note-taker assisted the investigator in recording the sessions using a voice recorder and written notes. ***Quantitative data*** were the scoring system results used to assess the understandability and actionability of the educational leaflets distributed to caregivers by HCPs. For scoring, researchers used the Patient Education Materials Assessment Tool for Printable Materials (PEMAT-P) (13). The tool provides two scores for each material: a score for understandability and a separate score for actionability. The researchers scored the education material on each item of the material content, excluding the non-applicable (NA) items. Each item was given either 1 point (agree) or 0 points (disagree) (Tables 1A, B). First, the total points for the material items were summed up to calculate the score. Then, the sum was divided by the total possible points: the number of items on which the material was rated, excluding the items classified as NA. After that, the result was divided by 100 to get a percentage (%). This percentage score is the understandability score or the actionability score of the material on the PEMAT; the more understandable or actionable the material, the higher the score (13).

**Table 1A:** Assessment of printed patient education materials provided to caregivers in CSPM (using PEMAT-P): understandability.

**Health education items:**	**Rating**
**Response options (rating): Agree: 1 Disagree: 0**	
**Contents**	
1. The material makes its purpose completely evident.	0
2. The material does not include information or content that distracts from its purpose	1
**Word choice and style**	
3. The material uses common everyday language.	0
4. Medical terms are used only to familiarize the audience with the terms and are defined if used.	1
5. The material uses the active voice.	0
**Use of numbers**	
6. Numbers appearing in the material are clear and easy to understand. (If no number = NA)	1
7. The material does not expect the user to perform calculations.	1
**Organization**	
8. The material breaks or “chunks” information into short sections. (Very short material = NA)	1
9. The material's sections have informative headers (very short material = NA)	1
10. The material presents information in a logical sequence.	1
11. The material provides a summary. (Very short material = NA)	0
**Layout and design**	
12. The material uses visual cues (e.g., arrows, boxes, bullets, bold, larger font, highlighting) to draw attention to key points.	NA
**Use of visual aids**	
13. The material uses visual aids whenever they could make content more easily understood (e.g., illustration of healthy portion size).	0
14. The material's visual aids reinforce rather than distract from the content. (No visual aids =NA)	NA
15. The material's visual aids have clear titles or captions. (No visual aids = NA)	NA
16. The material uses illustrations and photographs that are clear and uncluttered (No visual aids = NA)	NA
17. The material uses simple tables with short and clear row and column headings. (No Tables = NA)	0
Total points = 17; Total possible points = 13; Total achieved points = 7	
Understandability score= 7/13= 54%	

**Table 1B:** Assessment of printed patient education materials provided to caregivers in CSPM (using PEMAT-P): actionability.

**Health education items:**	**Rating**
**Response options (rating): Agree: 1 Disagree: 0**	
1. The material clearly identifies at least one action the user can take.	1
2. The material addresses the user directly when describing actions.	0
3. The material breaks down any action into manageable, explicit steps	0
4. The material provides a tangible tool (e.g., menu planners, checklists) whenever it could help the user take action.	1
5. The material provides simple instructions or examples of how to perform calculations. (No calculations = NA)	NA
6. The material explains how to use charts, graphs, tables, or diagrams to take action. (No charts, graphs, tables, or diagrams = NA)	NA
7. The material uses visual aids as much as possible rendering it easier to act on the instructions.	0
Total points = 7; Total possible points = 5; Total achieved points = 2	
Actionability score = 2/5 = 40%	

### Statistical analysis

#### For qualitative data

Data processing was based on the thematic content analysis technique (14, 15), which aims to get descriptions of the message content using a systematic and objective procedure. The thematic analysis is a three-stage analysis. The *first stage* involves understanding the idea through comprehensive and repeated readings of the data transcripts. *The second stage* is material exploration, which involves selecting the participants' statements and organizing them into categories (themes). *The third stage* deals with the processing and interpretation of results. The participants' quotations were used to clarify the meaning of the themes and summaries. Two investigators carried out the analysis. They read the transcript multiple times, made meaningful statements, and created themes and sub-themes.

#### For quantitative data

The PEMAT-P tool provides two scores for each material: a score for understandability and a separate score for actionability. The researchers scored the education material on each item of the material content, excluding the non-applicable (NA) items. Each item was given either 1 point (agree) or 0 points (disagree) (Tables 1A, B). First, the total points for the material items were summed up to calculate the score. Then, the sum was divided by the total possible points: the number of items on which the material was rated, excluding the items classified as NA. After that, the result was divided by 100 to get a percentage (%). This percentage score is the understandability score or the actionability score of the material on the PEMAT; the more understandable or actionable the material, the higher the score (13).

### Ethical considerations

The study protocol was revised and approved by the Medical Research Committee in the Public Health and Community Medicine Department. All the study participants were treated according to the Helsinki Declaration of biomedical ethics (16). Written informed consent from each participant was obtained after proper orientation regarding the study objectives. Data confidentiality and informant privacy were upheld throughout the whole study. For each participant, we used “I” (interviewee) followed by a number per the chronological order of the interviews (I01, I02, I03..., etc.). The necessary municipal and federal authorities approved the study to be carried out. Using the voice recorder was authorized, and transcriptions of the recordings were performed.

## Results

### Qualitative data analysis results

By analyzing the qualitative data derived from the interviews, the researchers came up with the following themes:

- Views of HCPs toward malnutrition problems

HCPs mentioned that “***the most frequent malnutrition problems that come to seeking*** ***medical advice were rickets, parasitism, underweight, kwashiorkor, and failure to*** ***thrive**.*”

Physicians mentioned that “***laboratory investigation was the best method to assess the*** ***nutritional status**.*”

To improve the child's nutritional status, all HCPs confirmed the importance of “***nutrition*** ***education, counseling, and provision of supplements such as iron and vitamin D***” during infancy.

- Barriers to providing nutrition care to children

HCPs affirmed that there are three types of barriers that restrict providing quality medical nutrition care.

#### Physician-centered barriers

- Limited opportunities for applied/clinical nutrition education in medical schools

Physicians mentioned that “***limited nutrition education in medical school and*** ***even in postgraduate studies (namely master's degree)***” is the major challenge for gaining an adequate medical knowledge. Physicians said that “***they learned the*** ***biochemistry of nutrition but not the basic nutrition knowledge needed to share with*** ***the patients**.*” They expressed dissatisfaction with their medical school education “***We believe that we are ill-qualified to provide nutrition advice in the clinical*** ***setting**.*”

Furthermore, physicians explained that the nutrition course they received was not applicable to patients. As a result, they were not prepared to counsel caregivers. Some HCPs felt their clinical rotations did not prepare them for the prevention or management of childhood malnutrition from the nutritional aspect due to the limited experience of tutors in clinical nutrition.

#### Health system-centered barriers

These include barriers related to the resources and process components of the health system.

- Inadequate capacity building of HCPs in the workplace

Physicians expressed their views of inadequate nutrition training programs for physicians: “***Training prepared us inadequately for work in low-resource settings**.*”* One* physician delineated his participation in training programs at the Faculty of Medicine, Cairo University. He said the first hospital training course started in June 2009 and included six sessions taught over 1 week. The training approach included theoretical educational sessions, practical training, demonstration exercises, group-based exercises, and case studies about breastfeeding. Another HCP received a nutrition course at the Ministry of Health in 2004, including four sessions taught over 2 days about growth monitoring. However, all respondents affirmed that they do not have access to regular refresher courses to reinforce their learning, provide practice demonstrations, and keep them up to date. In addition, they identified specific areas that are not satisfactorily covered in their nutrition education: nutrition counseling, infant and young children feeding, breastfeeding, and nutritional treatment of micronutrient deficiencies.

HCPs mentioned that training is significantly **infrequent**; that they hadn't received any nutrition care training in the past year. The most recent source of information they had about the contemporary infant feeding issues was the patients' handouts from the National Nutrition Institute. The only topic in which HCPs received training was exclusive breast feeding (EBF) where physicians and nurses have had similar exposure to training. Nurses attended more training than physicians. However, there is a rapid turnover of physicians and nurses, which makes such training needed continuously.

- Insufficient time to provide quality nutrition care

Physicians ascertained that they could not provide quality nutrition counseling in the CSPM malnutrition clinic due to the **high caseload** for the **small number of physicians and nurses**. Thus, they do not have enough time for growth monitoring or caregiver education on EBF, infant nutrition, sick children's nutrition, and pregnant women's nutrition. In addition, they added that there are too many patients per day, patient contact time is roughly 20–25 min long, and there are around 40 patients every day, except on Saturdays, on which there are even more patients.

Three HCPs said local women are always interested in hearing them talk and demonstrate rather than watching or reading visual materials independently. HCPs explained that with so little time spent with patients in the clinic, nutrition counseling is quite challenging. One physician said, “***In my opinion, there are relatively few things that doctors can do to help*** ***set up the malnutrition clinic because malnutrition is a complex problem that necessitates*** ***behavioral intervention. However, there is not enough time for that**,” and “**Physicians*** ***focus on immediate medical concerns instead of the more long-term concern of childhood*** ***nutrition**.*”

- Shortage in the workforce and lack of motivation

HCPs cited a shortage in staffing as a barrier to meeting their clients health and nutrition needs. They all mentioned, “***There has been no new recruitment of residents and*** ***nurses, no incentives or support in recognition of our hard work**.*” On the other hand, the low salary (1200 LE per month), insufficient opportunities for financial growth, and the lack of professional advancement increased the turnover rate of the already-appointed residents. Temporary contracts were used to hire all new doctors. The consensus among HCPs was that “***There is a need for more service*** ***providers**.*”

- Shortage in logistics and supplies

HCPs demarcated the scarcity of **visual PEM**, **resources for demonstrations** for nurses regarding infant feeding, especially breastfeeding charts, posters (particularly those showing local foods in food groups and demonstrating proper hygienic practices), information leaflets, booklets, and picture information cards. In general, the availability of PEM for caregivers was suboptimal for all areas of child health except for child feeding starting at 6 months up to 9 years of age.

Weighing scales (for babies and pregnant women), stature measuring boards (for length and height), oral rehydration solution (ORS), and infant formula milk for infants in particular categories (6–24 months) were available. However, **nutritional supplements** such as iron, zinc, folic acid, and vitamin A were suboptimal and coupled with a marked shortage of **PEM addressing micronutrient deficiencies**.

There were no CSPM-**specific guidelines** or national **protocols** for the nutritional management of malnourished children. Only educational and instructional leaflets from the National Nutrition Institute for complementary feeding are available.

#### Caregivers-centered barriers

- Low socioeconomic status (SES)

HCPs recognize that “***childhood malnutrition is a complicated problem influenced by*** ***numerous factors**.*” This concept makes HCPs understand that there are barriers facing caregivers face in trying to comply with nutritional advice. The most obvious barrier is the low SES status with subsequent limited access to healthy food and inability to recall the detailed medical advice provided.

Physicians mentioned that caregivers usually have limited nutrition knowledge, preventing them from selecting and preparing healthy complementary food. One HCP said, “***Many parents who are unaware of what is and is not healthy for their*** ***children**.*”

- Culture and context:

Recognizing that people had prior **specific cultural or traditionally-rooted beliefs is critical**. This included the belief that breastfeeding after getting pregnant could cause kwashiorkor in the breastfed child. The potential conflict between health workers' counseling and cultural beliefs and perceptions is more general, extending beyond nutrition issues, and has been well known.

### Suggestions of HCPs to improve performance in the malnutrition clinic

- Updating knowledge by continuing medical education (CME)

HCPs demanded more nutrition education, saying it could be provided through periodic rotations and seminars. One physician said, “***We are provided periodic lectures on asthma;*** ***why we do not have lectures on nutritional therapy for malnutrition? Not only to teach us*** ***about malnutrition epidemiology, but to show us some of the techniques and resources we*** ***could need in the future, as well as to instruct us on how to deal with patients in the real*** ***world**.*”

One physician said, “***To help parents improve their infants' nutrition, doctors must be*** ***aware of the specific advice they can give the caregivers regarding their infants'*** ***feeding—what, when, and how to feed***.” HCPs expressed a need for specific hints on preparing healthy food that could be shared with patients. HCPs stated that they required evidence-based nutrition expertise in addition to patient-friendly information. They valued recommendations based on scientific literature.

HCPs mentioned the most needed topics to emphasize in education: “breastfeeding, complementary feeding for 6- to 24-month-old children, counseling skills, and communication skills.”

They recommended getting continuous support and training through nutrition seminars and the introduction of software programs on nutrition counseling to facilitate and ensure proper quality performance of HCPs in malnutrition management, such as history taking, diagnosis, and nutrition education and counseling, including standardization of the nutrition counseling process. In addition, the curriculum of undergraduate training for medical and nursing students should include applied nutrition.

- Counseling skills

HCPs affirmed the need for “***more capacity-building in nutrition counseling***” as practical training on counseling caregivers is crucial for learning by doing.

Four out of five interviewees believed that physicians and nurses have the greatest need to understand nutrition education, communication, and counseling because they are in direct contact with the target groups for nutrition care (mothers and children). In Egypt, the public and patients usually seek nutritional advice and counseling from physicians, who often lack nutrition knowledge.

Regarding service delivery, there was a consensus that CSPM clinics needed to be strengthened by providing them with the required staffing for nutrition care services, nutrition training facilities (e.g., a working nutrition kitchen), and PEM to support both preventive and curative nutrition care. HCPs expressed a willingness to provide quality services if the necessary resources were made available and the issue of a large caseload was addressed. They also suggested increasing the availability of dieticians in childcare clinics, including malnutrition clinics.

Counseling was a challenging demand as counselors, on the one hand, try to simplify messages to make them easily understandable, and simultaneously provide mothers with sufficient information to make informed choices.

- Guidelines for efficient and effective nutrition care service

Guidelines must be developed on values that match the SES and culture of the clients. Disregarding those aspects have numerous and severe drawbacks, including infeasibility/inapplicability of the medical recommendations, confusion among both mothers and HCPs, and harmful feeding practices with diets that are inadequate for meeting the nutritional needs of the children.

### Quantitative data analysis results

The PEM provided to the caregivers at the malnutrition clinic was assessed using the PEMAT-P scoring tool regarding the understandability and actionability of the material.

The PEM had an understandability score of 54% and an actionability score of 40%, respectively (Tables 1A, B).

## Discussion

The current study explores the barriers to providing adequate nutrition care to children as perceived by the workforce (HCPs) at the CSPM malnutrition clinic which exists in a law-resource setting (i.e., has minimal budget allocations and serves a low-SES community). Although this clinic is logistically, financially, and legally affiliated with the MOH, it is located at the Children's Hospital of the Faculty of Medicine, Cairo University. It is considered a reference center to which cases diagnosed as malnutrition are referred from the hospital clinics. The CSPM malnutrition clinic is considered a model center for managing children's malnutrition using nutritional supplements and nutrition education to caregivers. Therefore, the conduction of the current study was crucial to help in decision-making for improving performance in the CSPM malnutrition clinic.

The study delineated that the most frequent malnutrition problems encountered in the studied clinic were nutritional deficiencies, namely rickets, underweight, kwashiorkor, and failure to thrive. This finding is consistent with the existing evidence in Egypt as per the Egypt Demographic and Health Survey (EDHS) and other local studies (12). The evidence also demonstrates that the significant factors, namely poverty, low maternal education level, and lack of health and nutrition awareness among parents, contributing to the high prevalence of such nutritional problems are related to the low SES of children caregivers (3, 17).

The study revealed the barriers to providing adequate nutrition care in three broad themes: physician-centered, health system-centered, and caregiver-centered.

The physician-centered barriers included limited education in clinical nutrition, mainly applied practical tactics, in medical school during both the undergraduate and postgraduate programs. HCPs perceived this barrier as the primary barrier. Many studies assessing the situation of nutrition education in medical schools have reported both undergraduate and postgraduate nutrition curricula as insufficient with a limited capacity of learners to identify cases of malnutrition or provide nutrition education for patients (18–20). However, other studies suggested that there is no need for more nutrition education in medical schools. The only requirement is to train future medical professionals to understand the role of nutrition in health and to encourage them to refer patients for nutrition counseling with a registered nutritionist or registered dietitian who is more qualified to provide dietary advice due to their greater education, training, and experience (21). Nevertheless, physicians who lack the necessary nutrition training could postpone making the initial diagnosis of malnutrition cases with the subsequent delay in the referral of cases to nutritionists or dieticians (7, 22).

The WHO defined the health system as all organizations, people, and actions whose primary purpose is to promote, restore, and maintain health (23). Therefore, as with any system, health system components are resources (workforce, finances, technology, and information, including updated standard of practice (SOP) and guidelines, process (the way of resource management), and output (range of provided services, e.g., how many of the clients are covered with services).^6^ In our study, health system-centered barriers are related to the resources and process components of the health system.

There was a workforce shortage due to insufficient HCPs regarding the high caseload. Hence, it was not only the lack of or inadequate skills of HCPs that hindered the provision of adequate nutrition counseling but also the insufficient time available for each patient. Individual needs should be considered when developing and communicating nutrition advice (24). Accordingly, a full assessment and understanding of the patient's psychosocial needs require roughly 15 min longer than simply comprehending the patient's initial complaint (22, 25). With the limited time concerning the high caseload, it is impossible to set aside that time for a needs analysis or dietary counseling.

Our findings also revealed the failure of the CSPM administration to recruit more HCPs, retain the competent HCPs, or upgrade the nutrition-related knowledge and service delivery skills of the existing HCPs, e.g., through providing updated SOP or guidelines and conducting periodic skill-based training. This situation led HCPs to try gaining updated information, infrequently and irregularly, from another healthcare institute, such as the National Nutrition Institute (NNI) at Cairo University.

In addition, HCPs complained of a lack of motivation due to the absence of appropriate incentives. Although some studies found that financial incentives did not cause long term behavior changes among physicians (26), yet, several other studies affirmed that the financial and otherwise incentives influence the behavior of HCPs in various ways, including adjustments to their output volume and effectiveness, as well as the type and standard of services they provide to clients (27, 28).

Nutritional supplementation with adequate nutrition education is an evidence-based essential package of nutrition interventions, particularly in low SES communities. To be effective, this package should be delivered during key life stages, such as during pregnancy and throughout childhood (29, 30). Hence, the lack of health technology and supplies such as nutritional supplements and PEM was viewed by the HCPs as a significant barrier to delivering adequate nutrition care. Moreover, the quality of the existing PEM was described by the HCPs as inadequate. This was also confirmed by the low PEM understandability and actionability scores (50 and 40%, respectively) which were far below the lowest recommended level (70%) necessary for patients to understand or act on the information they receive from the material (13). This is expected because the communication section of the MOH, which produces these PEMs, does not pre-test most of the health education materials or update the channels of conveying them. For instance, short educational videos that convey important health messages in a simple and practical form are not available at all at the CSPM.

Similar health system-centered barriers were stated by physicians in several studies, such as the pivotal study conducted by Kushner in 1995 and another study conducted 15 years later (31, 32). Those studies found that the most significant barriers to providing quality nutrition service were the lack of incentives and time followed by the lack of updated knowledge. However, in our study, HCPs first stated the lack of updated knowledge and refreshing training. This may be because Egypt's health sector workforce policy permits HCPs to work in both the public and private health sectors, allowing HCPs to operate their private clinics and increase their income.

Unfortunately, the insufficient resource component of the health system is a commonly encountered barrier in many countries (8, 31, 32) because healthcare funds are skewed mainly toward treatment, with little funds directed toward nutrition education programs or malnutrition prevention in general (33, 34). However, the insufficient resources barrier in child nutrition care is more encountered in low-and-middle-income countries and usually negatively impacts children under five the most. This is unlike in higher-income countries where children who are negatively impacted in nutrition care are those older than 5 years of age, usually hospitalized, with the barriers related mainly to the process component of the health system (35). Economic growth has been widely considered an effective tool for mitigating poverty and improving public health (4, 33, 36). In Egypt, even though the total health expenditure- as an absolute number- is increasing, the total health expenditure as a percentage of the gross domestic product is declining. Also, the Egyptian healthcare market is mainly based on out-of-pocket payments (the expenses for healthcare that are not reimbursed by insurance) (5, 8, 37). Thus, considering the progressively declining SES of the Egyptian population, the Egyptian healthcare system, specifically the preventive sector, whose budget is the primary source of funds allocated for child nutritional care in the public sector, is facing extreme financial challenges (5, 38). Cost-effectiveness (determining the expected gains and cost per gain) is central to the health system's success. Therefore, health system resources should be directed to health services after making cost-effective analyses to decide on proper resource allocation, e.g., allocating resources for nutrition care technology, such as nutritional supplements, and specific community groups (39, 40).

## Implications

The study focused on a significant public health problem in Egypt and most developing and underdeveloped countries, childhood malnutrition. This study tackled a crucial issue related to the crucial role of pediatric clinical nutrition services in overcoming such a problem. As well, the study provided valuable insight into barriers to providing adequate nutrition care to children. The identified barriers are essential when considering quality improvement of nutrition care practices for malnourished children.

## Strengths

First; the study was conducted in a model malnutrition clinic in a Children's Hospital, presenting a prototypical solution to overcome children's malnutrition problems and their impact on growth and development. Second; the study was of a qualitative research nature and thus provided an in-depth understanding of barriers hindering the provision of adequate clinical nutrition services and counseling. Third; using a specific approach for testing the PEM added another dimension to the study, as it raised the importance of capacity building in designing and testing the PEM. And fourthly; the clinical nutrition concept was raised in the study for specific pediatric clinics. The same concept could be applied to other medical clinics.

## Limitations

From our view, limitations of the study include that the study was conducted in one center, the only reference center for managing malnourished children. Additionally, as with any exploratory operations research, information derived from the study could not be generalized. However, the methodology used in the study could be generalized to be used in different medical settings. Furthermore, the views of the clients (caregivers) were not examined as it was beyond the scope of this study. Those views may have led to more understating of nutrition services' quality and barriers to adequate service. However, surprisingly, previous studies, such as one conducted in Bangladesh, showed that caregivers' satisfaction was above average despite the low quality of child nutritional services (27). The low expectations of the caregivers in the low SES communities could explain this. Similarly, a relatively high level of satisfaction could have been shown in this study for the same reason and because there are not many pediatric nutrition care clinics serving this district, making this clinic a unique one-of-a-kind center.

## Recommendations

To overcome barriers to providing adequate care in malnutrition clinics, there are multifaceted approaches directed to upgrading the resources and process components of the health system as follows: **Medical schools are encouraged to** integrate clinical nutrition in the pediatrics curriculum of undergraduate and postgraduate medical students and nursing students. Also, the specialty of clinical nutrition should be introduced into the masters and doctorate degrees and clinical nutrition training agenda should be included in the continuous medical education program to improve nutrition-related knowledge, counseling skills, and capacity building in designing and testing health education materials. Furthermore, marketing for the unique health and nutrition services provided to children in the nutrition clinics motivates HCPs to work in such clinics and encourages decision-makers to improve resources, especially regarding nutritional supplements, to such clinics. Regarding **MOH, we recommend the establishment of** pediatric clinical nutrition fellowship programs, introducing clinical nutrition clinics in the primary health care facilities and hospitals, increasing the production and availability of nutritional supplements, particularly for children's most common nutritional deficiency problems, and producing adequately designed and tested PEMs that suit the sociodemographic characteristics of the served communities.

## Conclusion

There are different barriers to adequate nutrition care for child malnutrition in low-resource healthcare settings. Mainly nutritional deficiencies. Most of the barriers were health system-related in the form of insufficient resources (shortage of workforce; concerning the high caseload, nutritional supplements, and PEMs) and inadequate management of resources (lack of skill-based training, lack of updated SOP and guidelines, and lack of properly designed PEMs that facilitate communication with the target caregivers).

## Data availability statement

The raw data supporting the conclusions of this article will be made available by the authors, without undue reservation.

## Ethics statement

The studies involving human participants were reviewed and approved by Faculty of Medicine, Cairo University (N-139-2022). The patients/participants provided their written informed consent to participate in this study.

## Author contributions

GE has made substantial contributions to the conception and design, analysis and interpretation of data, and writing the manuscript. MS has made substantial contributions to the acquisition of data and writing the manuscript. AT, SH, EA, and SI were involved in drafting the manuscript (Sections Methods and Results) and revising it carefully for important intellectual content and statistical analysis. All authors read and approved the final manuscript.

## Footnotes

^1^Available online at: https://www.who.int/news-room/fact.sheets/detail/malnutrition (accessed June 2, 2021).

^2^Available online at: https://www.who.int/publications/i/item/9789240025257 (accessed September 12, 2021).

^3^Available online at: https://www.who.int/publications/i/item/WHO-NMH-NHD-14.3 (accessed December 20, 2021).

^4^*UNICEF Report*. (2021). Available online at: https://www.who.int/publications/i/item/9789240025257 (accessed December 20, 2021).

^5^Available online at: https://egypt.un.org/en/sdgs/2 (accessed May 21, 2022).

^6^Available online at: https://healthmanager.ie/2011/03/the-differences-between-outputs-and-outcomes/ (accessed September 20, 2021).
