# Supplementary material for: Barriers to adequate nutrition care for child malnutrition in a low-resource setting: Perspectives of health care providers
Source: Front Public Health. 2023 Mar 9;11:1064837. doi: 10.3389/fpubh.2023.1064837 (PMC10034359; doi:10.3389/fpubh.2023.1064837)
Supplement: Supplementary file 1 [file Table_1.docx]

**Appendix 1: In-depth interview guide**

| Items | Interview guidelines |
| --- | --- |
| Baseline characteristics | • Role in the organization?  • Professional qualifications (education and training) |
| Current situation,  barriers, and  suggestions to  improve nutrition counseling | What are the most common malnutrition problems you encounter in the clinic? |
|  | Does your workplace have guidelines or follow national protocols for the nutritional management of malnourished children? |
|  | What are the challenges (constraints) you face in counseling caregivers on feeding options? |
|  | Do you have any suggestions for improving nutritional counseling? |
